# Anatomy and baseline histology of the hoof capsule, corium, and digital cushion in free-ranging southern giraffe (*Giraffa giraffa*)

**DOI:** 10.1371/journal.pone.0339972

**Published:** 2025-12-30

**Authors:** Liza Dadone, Steve Foxworth, Jacqueline Goedhals, Sushan Han, Priya Bapodra-Villaverde, Seng Wai Yap, Thato Radile, Francois Deacon

**Affiliations:** 1 Giraffe Veterinary Services, Colorado Springs, Colorado, United States of America; 2 Zoo Hoofstock Trim Program, Loveland, Colorado, United States of America; 3 Path Care Laboratories, Bloemfontein, South Africa; 4 Faculty of Health Sciences, Department of Anatomical Pathology, University of the Free State, Bloemfontein, South Africa; 5 Denver Zoo, Denver, Colorado, United States of America; 6 Columbus Zoo and Aquarium, Powell, Ohio, United States of America; 7 University of Wisconsin College of Veterinary Medicine, Madison, Wisconsin, United States of America; 8 Faculty of Natural and Agricultural Sciences, Department of Animal-, Wildlife- and Grassland Sciences, University of the Free State, Bloemfontein, South Africa; University of the Faroe Islands: Frodskaparsetur Foroya, FAROE ISLANDS

## Abstract

The front feet of six adult free-ranging southern giraffe *(Giraffa giraffa*) were opportunistically examined to characterize normal hoof anatomy, focusing on the corium (dermis), which provides vascular supply, metabolic support, and structural templates for the overlying epidermis that generates the keratinized hoof capsule. Gross dissection and histology identified two types of corium on the surface of the distal phalanx (Pd): laminae and papillae. On the parietal surface of Pd, laminae covered approximately its distal two-thirds and, as in other ruminants, secondary laminae were absent. Papillae varied regionally, with the longest and thickest located at the distal margins of Pd. On the solar surface, horn tubules were oriented obliquely in a palmar-proximal to dorso-distal direction. Within the hoof, the digital cushion consisted of a proximal adipose-rich region and a distal fibroelastic region. Histological findings were unremarkable and supported gross observations of normal anatomy. Examination of Pd and the navicular (distal sesamoid) regions revealed no evidence of pedal osteitis, navicular pathology, laminitis, or other lesions. These data provide a reference for normal giraffe foot anatomy and histology. Improved understanding of the corium and associated structures that support hoof capsule growth may inform preventative hoof care, reduce risk of overgrowth, and assist in managing lameness in both zoo-housed and free-ranging giraffe.

## Introduction

Hoof overgrowth and associated lameness are well-recognized health concerns in zoo-housed giraffe (*Giraffa spp.*) [1–4]. Hoof capsule overgrowth alters weight-bearing surfaces and joint movements in the distal limb, predisposing zoo-housed giraffe to degenerative conditions such as osteoarthritis, pedal osteitis, fractures of the distal phalanx (Pd; P3), navicular disease, and ligament injuries [3–9].

In giraffe and other ungulates, the hoof capsule protects the distal limb and supports body weight [9–12].The hoof capsule comprises the wall, sole, and heel, and grows continuously to compensate for wear and mechanical stress, serving as a structural barrier, shock absorber, and load-distributing surface [10–11]. The capsule itself is composed of keratinized epidermis, produced by basal epithelial cells. These cells form the deepest layer of the epidermis, directly overlying the corium (dermis). The corium provides vascular supply, metabolic support, and structural dermal templates (papillae and laminae) that interdigitate with basal epidermal cells, enabling horn tubule formation and anchoring the hoof wall to the distal phalanx to distribute mechanical forces during locomotion [10,13]. Unlike papillary regions at the coronet and sole, epidermal laminae are not the primary sites of horn production. Thus, while the corium is dermis rather than epidermis, it is indispensable in sustaining keratinization and maintaining the structural integrity of the hoof capsule [13,14]. Damage to the corium from trauma, inflammation, or ischemia can result in hoof deformity and loss of internal structural integrity [10,13].

The digital cushion, located on the caudodistal aspect of the foot, functions as a dynamic load distributor [10]. In artiodactyls, each digit contains a digital cushion, a deep digital flexor tendon (DDFT), and a single distal sesamoid bone (navicular bone), and the paired digits together form the foot. The digital cushion is composed of both fibrous and adipose tissues and lies palmar/plantar to the DDFT and distal sesamoid bone, deep to the hoof capsule at the heel/sole margin. This structure helps dissipate forces during locomotion, stabilizes the foot, and protects the DDFT, navicular bone, and corium. The digital cushion also supports venous return from the distal limb through its cyclical compression and relaxation during weight-bearing. In other megavertebrates such as rhinoceros and elephants, foot pathologies often involve degenerative changes to the digital cushion [15–17], suggesting that mechanical overload or altered biomechanics may predispose to structural compromise in this region.

Ungulate foot health is strongly influenced by substrate. In feral horses, hard, abrasive terrain combined with long travel distances produces shorter, less flared hoof walls, whereas softer substrates promote longer, flared growth [18–21]. Similar patterns are seen in managed ruminants, where housing systems that limit natural wear frequently exacerbate overgrowth. Dairy goats kept on uniform flooring developed excessive hoof growth despite trimming every four months, resulting in poor conformation and secondary joint changes, particularly in the hind limbs [22]^.^ In cattle, flooring design alters hoof health; cows maintained on rubber-slatted flooring showed fewer cases of dermatitis, heel horn erosion, and sole lesions than those housed on solid floors [23]^.^ In giraffe, natural substrates such as sand and rocky terrain promote normal wear, while uniform zoo substrates can predispose to hoof overgrowth. Consistent with these substrate-related effects, prior studies have documented differences in foot shape, radiographic anatomy, and foot pathologies between free-ranging and zoo-housed giraffe [24]. Zoo husbandry guidelines also emphasize substrate management as a practical tool for prevention. However, in managed settings, natural substrates alone are often insufficient. Regular hoof trimming is also important to maintain foot health [9].

Beyond substrate, multiple intrinsic and extrinsic factors may contribute to hoof overgrowth and lameness in giraffe. In managed care, these include genetics-based conformation abnormalities, variations in hoof growth rate, diet, enclosure size, limited exercise opportunities, and metabolic health. In free-ranging populations, risks include snare injuries, nutritional imbalances, and terrain variability. Habitat fragmentation and resulting inbreeding on private ranches (≈ 12,000 in South Africa) [25] may further increase susceptibility to foot pathology.

To establish a baseline for normal distal-limb anatomy, we conducted detailed gross and histological examinations of the front feet from six free-ranging southern giraffe (*Giraffa giraffa*). These data will help distinguish natural structural variation from disease-related changes and inform preventive hoof-care strategies for both zoo-managed and wild giraffe.

## Materials and methods

### Ethics statement

Specimen collection and use were approved by the University of the Free State Animal Ethics Committee (UFS-AED2023/0082) with provincial authorization (DESTEA permit 202404000015454). All giraffe in the study had died prior to project conception; distal limb specimens were opportunistically collected for research purposes and stored frozen at −20°C.

### Animals

In August 2024, four male and two female giraffe (*Giraffa giraffa*) were culled by a private reserve in South Africa as part of annual habitat management efforts to support carrying capacity. All six were classified as adults based on body size and dentition and appeared to be in overall good health, although exact chronological ages were not available. The giraffe inhabited a diverse environment spanning approximately 37,000 hectares (370 km^2^), consisting primarily of savanna grasslands with areas of sandy and rocky terrain. For capsule reflection/photography, the left front and right hind feet from one adult male were studied. Histology and the longitudinal section analyses were performed on the medial digit of the right front foot from each of the six giraffe.

### Gross dissection and capsule removal

**Fig 1:**
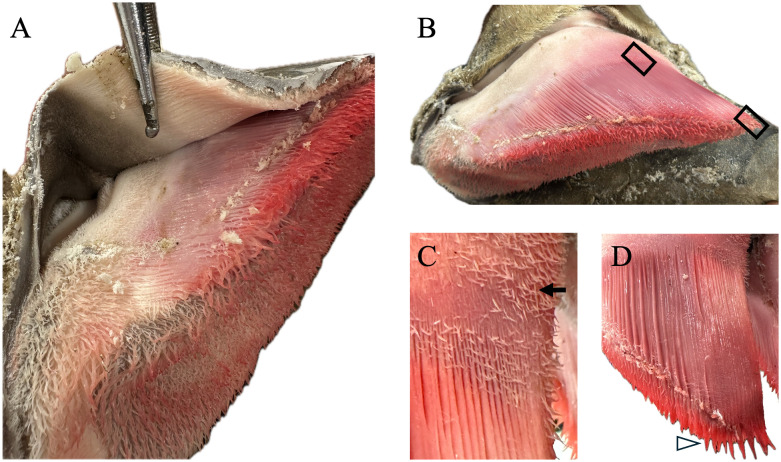
Giraffe hoof capsule and corium. **(A)** Reflection of the hoof wall reveals both insensitive and sensitive tissues, including the interface between the hoof capsule and corium. **(B)** After removal of the hoof wall, the parietal surface of the distal phalanx (Pd) corium displays its normal proximal-to-distal organization. The more proximal region (outlined, left) corresponds to the coronary cushion and contains short papillae. Distally, these papillae transition into long, parallel primary laminae (outlined, right), forming the laminar corium. A gradual transition zone occurs naturally between these regions. **(C)** Higher magnification of the coronary band shows fine, elongated papillae (←), while the adjacent laminar region displays broad, elongated laminae (Δ). **(D)** At the toe tip and lateral margins of the digit, the corium consists of long, broad papillae (Δ), consistent with load-bearing areas.

Six months later, the left front and right hind feet from that adult male were thawed and heat-shocked by immersion in boiling water for 6.5–8.5 minutes. The hoof wall was then cut vertically in several locations with a Dremel tool to the level of the corium to facilitate the removal of the hoof capsule and to visualize sensitive and insensitive structures (Fig 1).

### Histological sampling and processing

**Fig 2:**
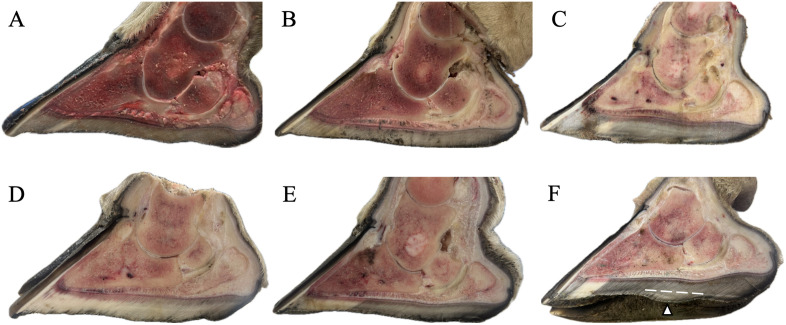
Sagittal sections of giraffe right front foot medial digits showing normal and abnormal sole alignment. Sagittal (parasagittal) sections are shown from four male giraffe **(A-D)** and two female giraffe **(E, F)**. In most feet **(A-E)**, the solar surface of the hoof capsule is approximately parallel to the distal margin of distal phalanx (Pd). In contrast, foot F shows localized caudal sole overgrowth (Δ). The dotted line indicates the expected plane of the solar surface in parallel with Pd; all capsule growth distal to this line represents overgrowth, with a distinct bulge visible distally in the sole. This panel is oriented obliquely to emphasize both the normal concavity of the sole and the abnormal caudal overgrowth. Feet A and B had been thawed before dissection, then re-frozen, and showed generalized hemorrhage on sectioning.

**Fig 3:**
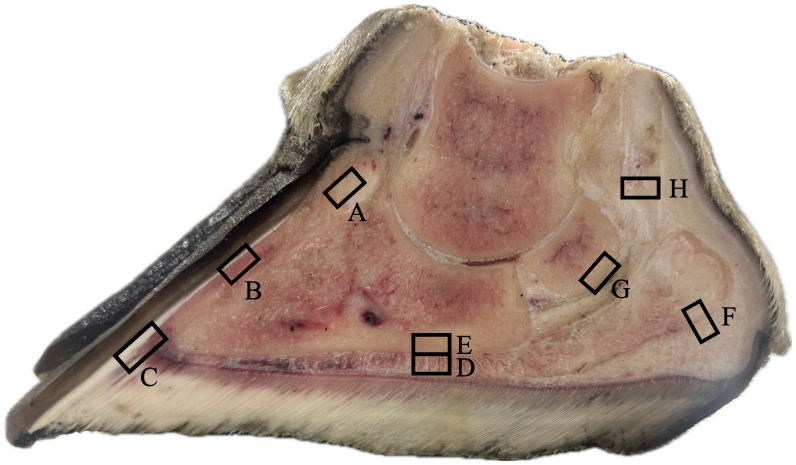
Histology sampling sites in the medial digit of a giraffe’s front foot. Corium samples were collected from: **(A)** coronary cushion, **(B)** laminae, **(C)** toe tip, and **(D)** caudal sole. Additional samples included: **(E)** solar surface of the distal phalanx (Pd), **(F)** digital cushion, **(G)** distal sesamoid bone, and **(H)** deep digital flexor tendon.

The medial digit of the right front foot for each giraffe was transected longitudinally with a band saw to evaluate the distal limb and hoof capsule (Fig 2). Samples for histology were collected from the corium, digital cushion, Pd, distal sesamoid bone, and DDFT, and were preserved in 10% neutral buffered formalin (Fig 3). Corium samples were collected from the coronary region, the laminae along the dorsal Pd surface, the distal tip of Pd, and the solar surface adjacent to the articular surface of the distal interphalangeal joint. Digital cushion samples included both proximal and distal compartments. All tissues were processed using standard histological techniques and stained with hematoxylin and eosin (H&E) for microscopic examination.

## Results

### Hoof capsule: solar morphology

**Fig 4:**
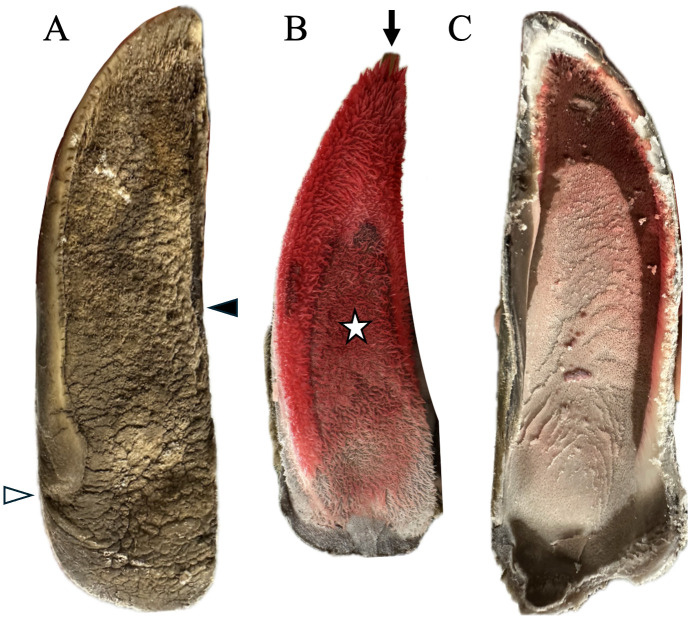
Solar surfaces of the hoof capsule and underlying corium in a giraffe. **(A)** The solar surface of the hoof capsule shows a relatively long peripheral wall and a centrally concave sole, with the heel and hoof wall serving as initial points of ground contact. The interdigital (**➤**) and caudal (Δ) hoof wall terminations define the borders of the caudal sole. **(B)** The exposed corium on the palmar/plantar surface of the foot also exhibits a central concavity. The papillae at the toe tip (←) and wall margins are longer, thicker, and darker red compared with the central sole, which contains shorter papillae (☆). **(C)** Close-up view of the paler corium of the central sole region corresponding to the ⋆ area in panel B, where the corium remains attached to the solar surface of the distal phalanx (Pd). In this region papillary height is reduced compared with the darker, more robust papillae at the toe tip and wall margins. The view is oriented from distal to proximal, analogous to panel A but with the epidermal sole removed.

On the solar surface of the hoof capsule, the hoof wall along the periphery of the foot was relatively long, with a concave central sole (Fig 4). Following removal of the hoof capsule, the underlying corium was characterized by longer papillae along the peripheral margins and toe tip, and shorter papillae within the central arch region of the sole. The corresponding internal surface of the hoof capsule had fenestrations and appeared darker red. In one specimen (Fig 2F), the hoof capsule extended distal to the expected solar plane parallel with the Pd, forming a caudal bulge consistent with localized sole overgrowth. Relative sole thickness at the toe tip, caudal sole, and heel are also visible in this cross-sectional image.

### Corium architecture and regional variation

After hoof capsule removal, the underlying corium was visible and showed distinct regional variation. Two types of corium were identified: corium papillae and corium laminae (Fig 1). On the parietal surface of Pd, the proximal one-third corresponded to the coronary cushion and consisted of fine papillae, and the distal two-thirds were composed of broader, longer laminae.

### Papillary morphology by location (toe, wall margins, central sole)

**Fig 5:**
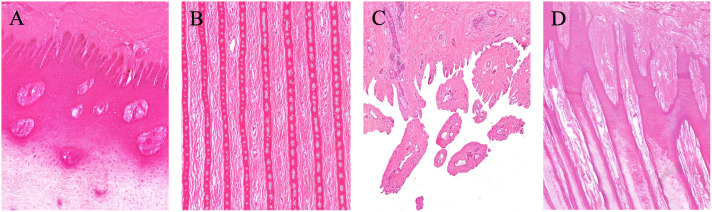
Corium histology in the giraffe front foot. The corium interdigitates with the hoof capsule through both papillary and laminar connections. **(A)** Coronary cushion: the corium forms papillae (H&E stain, 9.4x). **(B)** Dorsal hoof wall: laminar connections are present without secondary laminae or evidence of laminitis (H&E stain, 10.3x). **(C)** Toe tip: elongated papillae are present (H&E stain, 6x). **(D)** Caudal sole: elongated papillae are also present (H&E stain, 2.5x).

Regional variation in corium color and papillary morphology was also evident. Along the toe tip and wall margins, the corium was darker red with longer, more robust papillae, while the central sole corium was paler with shorter papillae (Fig 4). Papillae were shortest and narrowest near the coronary cushion (Fig 1C), became longer and thicker in the central sole (Fig 4B), and reached their longest and thickest at the distal margins of Pd (Fig 4B). Histology confirmed these regional variations in corium (Fig 5). Secondary epidermal laminae were not observed in the samples examined. No evidence of laminitis, pedal osteitis, navicular pathology, or other lesions was detected.

### Sole thickness and horn tubule orientation

**Fig 6:**
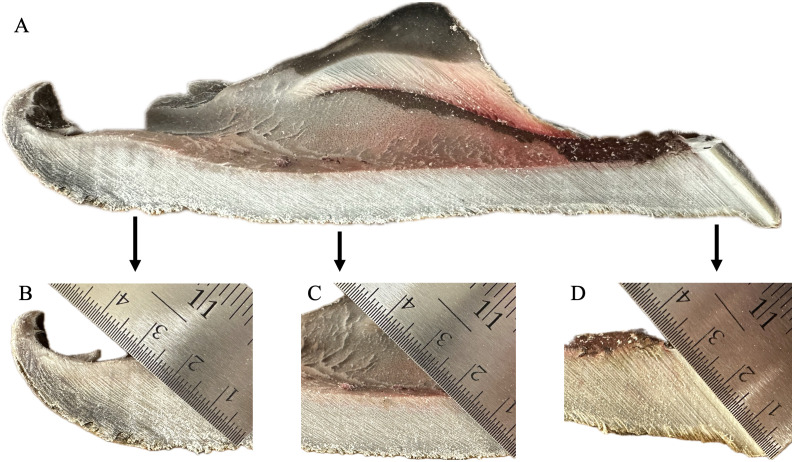
Sagittal section of the giraffe hoof capsule sole with regional close-ups. **(A)** Longitudinal section of the sole from heel to toe (toe tip oriented to the right). The sole is thickest beneath the toe tip and heel, and thinner across the central and caudal sole regions. Tubules within the sole are arranged diagonally, angling dorso-distally toward the tip of the hoof. **(B)** Heel region: ruler demonstrates sole thickness and diagonal tubule orientation. **(C)** Central sole region: thinner sole with consistent diagonal tubule orientation. **(D)** Toe tip region: ruler indicates greatest thickness and oblique tubule orientation.

On longitudinal sectioning of the hoof capsule’s solar surface, the sole was thickest beneath the toe tip and heel, and thinner across the central sole region (Fig 6A). Magnified insets show regional variation, with the heel (Fig 6B) and toe tip (Fig 6D) having the greatest thickness, and the central sole (Fig 6C) showing reduced thickness. In all regions, keratin tubules were oriented obliquely, angling dorso-distally toward the tip of the hoof.

### Digital cushion architecture

**Fig 7:**
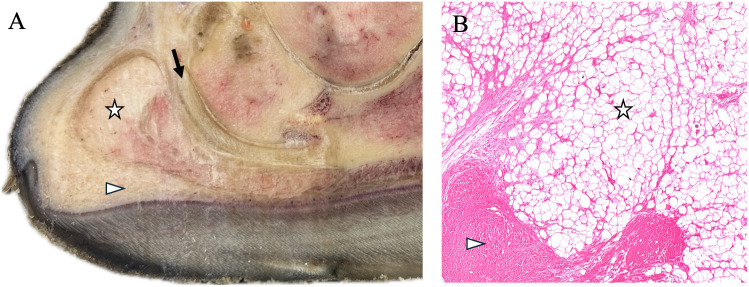
Cross-sectional anatomy and histology of the giraffe digital cushion. **(A)** Gross transverse section of the distal limb showing two distinct regions of the digital cushion: a proximal soft region (☆) composed of adipose tissue and a distal firm region (Δ) composed of dense connective tissue. The proximal region lies palmar to the deep digital flexor tendon (→) and extends distally beyond its terminal attachment, adjacent to the distal sesamoid bone of the digit. **(B)** Histological section (H&E stain, 4.9X) of the same regions confirms this distinction, with the proximal portion (☆) consisting of adipose tissue and the distal portion (Δ) composed of dense fibrous connective tissue.

The digital cushion consisted of two distinct regions: a proximal soft region and a distal firm region (Fig 7). The proximal portion lay palmar to the DDFT and extended distally beyond its terminal attachment, adjacent to the distal sesamoid bone of the digit. On histology, the proximal and distal regions were composed of adipose tissue and dense fibrous connective tissue, respectively.

### Screening for lesions (gross and histologic)

**Fig 8:**
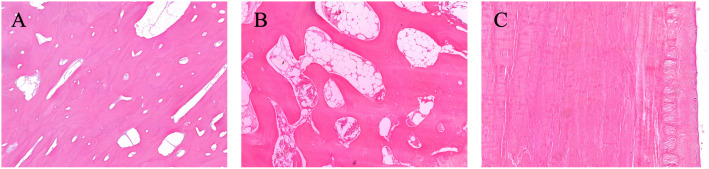
Histology of giraffe distal limb structures. Histological sections show: **(A)** Distal phalanx (Pd) near the caudal sole (H&E, 6.2x); **(B)** Pd (H&E, 6.3x); and **(C)** deep digital flexor tendon (DDFT (H&E, 8.7x). No significant pathologic changes were identified in these tissues.

Both grossly and histologically, no significant pathologies were identified in the Pd, distal sesamoid bone, or DDFT of any of the examined specimens (Fig 8). This included no evidence of pedal osteitis, Pd fractures, distal sesamoid cysts or lytic changes, or fibrotic changes of the DDFT. Grossly, variable degrees of localized or diffuse hemorrhage were observed in the distal limbs, particularly in the two feet that had been previously thawed (Fig 2A, 2B).

## Discussion

### Overview and context

This study provides the first comprehensive anatomical description of the hoof capsule, corium, and digital cushion of free-ranging southern giraffe, including gross and histological findings. These structures support hoof growth and mechanical load distribution and may differ from those in managed zoo populations.

### Hoof loading and horn/corium architecture

In the naturally worn feet of these free-ranging giraffe, initial ground-contact surfaces were observed along the peripheral hoof wall and heel, with a concave central sole that participates dynamically in load-bearing but typically carries lower peak loads. These observations are consistent with prior descriptions of wild giraffe feet [24] and suggest a likely weight-bearing pattern, although biomechanical measurements were not directly performed in this study. This contrasts with zoo-managed giraffe, which often develop sole flattening and hoof overgrowth [9]. The localized caudal sole overgrowth observed in one free-ranging giraffe (Fig 2F) highlights how deviations from the normal alignment between hoof capsule and the distal phalanx can be detected and corrected, and supports the use of this alignment as a trimming landmark in managed giraffe. Differences in hoof capsule shape and sole thickness between free-ranging and zoo-managed giraffe likely reflect the combined influences of substrate, exercise, and husbandry. In particular, uniform zoo flooring and exhibit habitats might limit natural wear and contribute to overgrowth, whereas free-ranging giraffe on variable terrains experience more balanced wear patterns [9,24]. These baseline data from naturally worn feet provide a useful reference point for evaluating how environmental substrates interact with hoof growth and trimming practices in managed populations.

Changes to sole thickness in zoo giraffe, and the resulting redistribution of weight across the foot, may contribute to vascular compromise, corium compression, and progressive degenerative changes of distal limb structures, leading to chronic lameness. These hypotheses warrant further study, although current recommendations for managed giraffe already emphasize routine hoof evaluations and corrective trimming to limit hoof overgrowth.

In these free-ranging giraffe, the external contour of the hoof capsule ran nearly parallel to the solar surface of Pd, with only a narrow horn layer separating the two. This near-parallel alignment—already used as a trimming landmark in managed giraffe [9]—supports its utility for gauging excess sole depth and early capsule distortion.

Regional differences in sole thickness and tubule architecture further reflected functional loading. The heel (Fig 6B) and toe tip (Fig 6D) had the greatest horn depth, while the central sole was thinner (Fig 6C), consistent with lower peak loads in that region. As these data were derived from a small sample set, they should be regarded as preliminary but provide useful insight into potential functional variations across the sole. In all areas, keratin tubules were oriented obliquely from the palmar-proximal surface toward the dorso-distal toe tip (Fig 6A). Tubules were longest beneath the toe tip and heels, a structural arrangement that likely aids in distributing load during weight-bearing and breakover. This pattern is consistent with prior studies in wild giraffe, where paint transfer experiments demonstrated initial ground contact at the toe tip, both heels, and along the hoof walls [24].

Two types of corium were identified within the hoof capsule: papillary and laminar, similar to descriptions in equine hoof anatomy [10,13]. Papillae were longest and broadest beneath the toe tip and heel, where wall thickness and load are greatest, and shortest near the coronary cushion. Laminar corium lined roughly the distal two-thirds of the parietal surface of Pd. In hoof-wall formation, the corium contributes vascular dermal papillae and laminae that support and organize growth at the dermal-epidermal junction, whereas the overlying epidermal keratinocytes produce the keratinized horn [10,13]. This epidermal-producing/dermal-supporting model at the dermal-epidermal junction is well established in equine sources and is likewise documented in bovine literature. [10,13,26,27]. Consistent with this, equine BrdU-labeling studies show minimal proliferation in distal laminae compared with the coronet/solar papillae, indicating that laminae function primarily in attachment/suspension rather than hoof-wall production [14]. The absence of secondary laminae is consistent with normal anatomy in artiodactyl species [11,12]. Histological screening did not reveal laminitis, pedal osteitis, or navicular pathology, providing further evidence that the sampled feet were structurally normal.

Regional differences in papillary morphology and appearance were evident, with longer, more robust papillae and a darker hue at the toe tip and wall margins, and shorter, paler papillae in the central sole (Fig 4). This pattern is consistent with greater vascular supply and horn-production demand in zones that experience higher and/or more sustained peak loading (toe and wall margins), whereas the concave central sole also participates in load bearing dynamically during stance and breakover but typically carries lower peak loads, producing thinner horn. Because the specimens were frozen (and some previously thawed), color intensity is likely influenced or accentuated by postmortem/freeze-thaw effects; therefore, our interpretation relies primarily on papillary size/architecture and their distribution relative to known loading patterns rather than color alone. Taken together, the consistency between papillary morphology, their location in known load-bearing areas, and prior ungulate literature supports true regional functional specialization rather than artifact [13,27,28].

### Digital cushion architecture and functional implications

The digital cushion was composed of two distinct tissue compartments: a proximal adipose-rich region and a distal fibroelastic zone extending toward the solar surface. This arrangement is similar to that of other ungulates, including domestic artiodactyls [11,12]. rhinoceros, [15] and elephants [17], and is hypothesized to aid in load distribution, shock absorption, and blood perfusion within the foot, and to support the DDFT. Extension of the digital cushion toward the sole may enhance protection of the DDFT, and degeneration of this region secondary to solar overgrowth could contribute to hoof pathology in zoo giraffe. Thinning of the digital cushion in dairy cattle has been associated with increased risk of lameness [29], and similar associations should be investigated in giraffe. In the samples examined, no histologic abnormalities were detected, although the limited number and scope of samples means that localized pathology could have been missed.

These findings provide a baseline reference for normal giraffe hoof anatomy and highlight structural adaptations for weight-bearing. They may help inform preventive hoof-care strategies for managed giraffe, although direct application to clinical practice will benefit from further biomechanical and pathological studies.

### Limitations

Several limitations should be considered. Giraffe were included opportunistically, and while tissues were well preserved, freezing artifacts and variation in hoof wear may have influenced observations. Histology was limited to specific planes and regions, and lesions in unsampled areas could have been missed. Only the medial digits of the front feet were examined; anatomy and loading patterns may differ between medial and lateral digits, and between fore- and hindlimbs. Exact chronological ages were not available, though all giraffe were adults, which limits fine-scale comparisons with zoo-housed populations where ages are documented. Measurements of sole thickness were based on relative comparisons visible in cross-sectional images (Fig 2) rather than standardized quantitative datasets. Absence of lesions such as pedal osteitis or Pd fractures cannot be confirmed without advanced imaging such as radiographs or CT.

## Conclusion

This study provides foundational anatomical data on the giraffe hoof capsule, corium, and digital cushion. Key findings include the regional variation in corium structures, diagonal tubule orientation in the sole, and dual-compartment structure of the digital cushion. These findings provide a reference for healthy hoof anatomy in free-ranging giraffe.

These insights may inform the development of hoof trimming practices and support early recognition of pathology in managed giraffe, contributing to improved wellbeing and conservation strategies for both zoo-housed and free-ranging populations. Further research into distal limb biomechanics, hoof growth under varying environmental conditions, and long-term effects of chronic changes to hoof-capsule shape will strengthen hoof management strategies and promote giraffe health globally.
